# Dermatological emergencies: a Moroccan retrospective case series over a period of two years

**DOI:** 10.11604/pamj.2022.41.348.28801

**Published:** 2022-04-29

**Authors:** Yasmina El Arabi, Fouzia Hali, Hayat Dahbi Skali, Soumiya Chiheb

**Affiliations:** 1 Department of Dermatology, Ibn Rochd University Hospital, Casablanca, Morocco

**Keywords:** Emergencies, skin diseases, drug eruptions

## Abstract

**Introduction:**

a dermatological emergency is defined as an acute dermatosis evolving since less than 5 days and being life or functional threatening. The main objective of this study was to describe the epidemiological and clinical profile of patients seen for a dermatological emergency.

**Methods:**

this is a retrospective case series, carried out over a period of two years [May 2018 - May 2020], including all the patients seen in the Dermatology Department for a true dermatological emergency. The descriptive analysis was carried out using Excel software.

**Results:**

a total of 843 patients were collected. The mean age was 46.95 years, with a standard deviation of 15.69 and a slight male predominance (n=448). There were 709 adults and 134 children. The majority of patients came from central emergencies (n=451). The pathologies seen were in order of frequency: Infectious dermatoses (n=469) dominated by erysipelas in adults (n=302) and viral dermatoses in children (n=47); drug-induced skin reactions (n=160); inflammatory dermatoses (n=113) including erythroderma (n=36), urticaria (n=32), vasculitis (n=25), and erythema multiform (n=20); autoimmune bullous dermatoses (n=74); and physical skin diseases (n=27). Other specialists´ advice was needed for 231 patients. Biological involvement and imaging were required in respectively 536 and 421 cases. Only 235 required hospital admission, while the others needed an ambulatory care.

**Conclusion:**

the pathologies seen in the dermatological emergency unit were dominated by infectious dermatoses, suggesting elaborating a medical program to improve the non-dermatologist physicians' knowledge about them.

## Introduction

Skin disorders represent 4-8% of all the pathologies seen in emergency care units, with only 30% being true emergencies [1]. The latter are defined as an acute dermatosis evolving since less than 5 days and being life or functional threatening [2]. They encompass infectious dermatoses, cutaneous drug reactions, inflammatory skin diseases, autoimmune bullous dermatoses, and some physical skin diseases [3]. However, many patients who seek medical care in the emergency department don´t represent real urgent cases. Despite their high prevalence, only few studies assessed the spectrum of skin pathologies leading to emergency consultations. The main objective of our study was to describe the epidemiological and clinical profile of patients seen in the dermatology department of the University Hospital Center of Casablanca for a dermatological emergency.

## Methods

**Study design and setting:** this is a retrospective case series study, carried out over a period of two years [May 2018 - May 2020], in the emergency unit of the Dermatology Department of Ibn Rochd University Hospital of Casablanca.

**Study population:** we included all the patients seen for a true dermatological emergency. Patients with incomplete data and those who consulted for non-true dermatological emergencies were excluded from the study.

**Data collection:** the data were collected using an analysis grid filled from the patients´ register. The data collected were: age, gender, referral mode, reason for admission, diagnosis, para clinical exams, therapeutic, and evolution. The diagnostics were grouped as infectious dermatoses, cutaneous drug reactions, inflammatory skin diseases, autoimmune bullous dermatoses, and physical skin diseases.

**Descriptive analysis:** the descriptive analysis was done using Excel Software. Quantitative variables were expressed as mean and qualitative variables as frequency or percentage.

## Results

**Figure 1:**
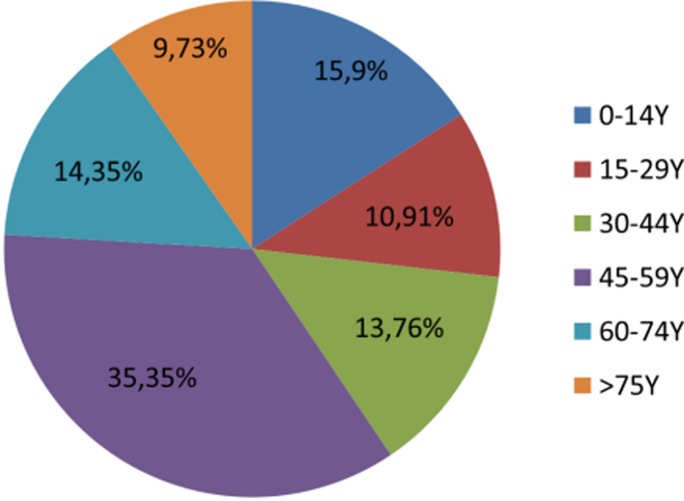
age distribution

From a total of 2912 patients seen in the dermatology emergency department, 843 presented a real urgent dermatological problem, and were included in the study. Out of those 843 patients, 709 were adults (84.1%) and 134 were children (15.9%). The mean age of patients was 46.95 years, with a standard deviation of 15.69. Age distribution is shown in Figure 1. The most represented age group was [45-59 years] (35.35%). A slight male predominance was noted, with 53.2% (n=448).

Four hundred forty-eight patients came to our unscheduled emergency consultation from central emergencies (53.2%), 155 patients from private doctors (18.3%), 110 patients from paediatric emergencies (13.1%), 70 patients from ophthalmological emergencies (8.3%), and 60 patients from gynaecological emergencies (7.1%). The clinical manifestations were in order of frequency: infectious dermatoses in 469 patients (55.63%) (Table 1), drug-induced skin reactions in 160 patients (18.98%) (Table 2), inflammatory dermatoses in 113 patients (13.4%) (Table 3), autoimmune bullous dermatoses in 74 patients (8.78%) (Table 4) and physical skin diseases in 27 patients (3.2%) (Table 5). Other specialists´ advice was needed for 231 patients (27.4%).

**Table 1:** distribution of infectious dermatoses

Infectious dermatoses	Prevalence		
	Adults (n)	Children (n)	Total (n)
Erysipelas	302 (35.82%)	0 (0%)	302 (0%)
Necrotizing cellulitis	14 (1.66%)	0 (0%)	14 (1.66%)
Herpes zoster	63 (7.43%)	3 (0.36%)	66 (7.79%)
Varicella	10 (1.19%)	14 (1.66%)	24 (2.85%)
Herpetic gingiva-stomatitis	10 (1.19%)	18 (2.13%)	28 (3.32%)
Phlegmon	6 (0.71%)	0 (0%)	6 (0.71%)
Ecthyma	6 (0.71%)	5 (0.59%)	11 (1.3%)
Measles	0 (0%)	6 (0.71%)	6 (0.71%)
Viral rash	0 (0%)	6 (0.71%)	6 (0.71%)
Staphylococcal epidermolysis	0 (0%)	6 (0.71%)	6 (0.71%)
Total (n)	411 (48.75%)	58 (6.88%)	469 (55.63%)

**Table 2:** distribution of drug-induced skin reactions

Drug-induced skin reactions	Prevalence		
	Adults (n)	Children (n)	Total (n)
Simple maculopapular exanthema	68 (8.1%)	40 (4.74%)	108 (12.81%)
Drug reaction with eosinophilia and systemic symptoms	24 (2.85%)	0 (0%)	24 (2.85%)
Acute generalized exanthematic pustulosis	10 (1.19%)	0 (0%)	10 (1.19%)
Lyell’s syndrome	8 (0.95%)	0 (0%)	8 (0.95%)
Stevens-Johnson’s syndrome	4 (0.47%)	6 (0.71%)	10 (1.19%)
Total (n)	114 (13.52%)	46 (6.45%)	160 (18.98%)

**Table 3:** distribution of inflammatory skin diseases

Inflammatory skin diseases	Prevalence		
	Adults (n)	Children (n)	Total (n)
Urticaria	26 (3.08%)	6 (0.71%)	32 (3.79%)
Erythroderma	34 (4.03%)	2 (0.24%)	36 (4.27%)
Erythema multiforme	10 (1.19%)	10 (1.19%)	20 (2.38%)
Vasculitis	17 (2.02%)	8 (0.95%)	25 (2.96%)
Total (n)	0 (0%)	0 (0%)	**113 (13.4%)**

**Table 4:** distribution of autoimmune bullous dermatoses in adults

Autoimmune bullous dermatoses	Prevalence (n)
Pemphigus	39 (4.63%)
Bullous pemphigoid	29 (3.44%)
Pemphigoid gestationis	4 (0.47%)
Cicatricial pemphigoid	2 (0.24%)
Total	**74 (8.78%)**

**Table 5:** distribution of physical skin diseases

Physical skin diseases	Prevalence		
	Adults (n)	Children (n)	Total (n)
Digital necrosis	6 (0.71%)	0 (0%)	6 (0.71%)
Wound	3 (0.36%)	2 (0.24%)	5 (0.6%)
Burn	14 (1.66%)	2 (0.24%)	16 (1.9%)
Total (n)	23 (2.73%)	4 (0.47%)	**27 (3.2%)**

Biological involvement and imaging were required in respectively 536 patients (63.58%) and 421 patients (49.94%). Among patients seen in the emergency unit, only 235 required hospitalization (27.88%), mostly for erysipelas or drug-induced skin reactions, while the others only needed an ambulatory care.

## Discussion

The objective of our study was to determine the epidemiological and clinical profile of patients who consulted for a real dermatological emergency. We noted that many patients consulted in the emergency department for a non-urgent dermatosis that could be managed in ambulatory. As for real dermatological emergencies, they were dominated by infectious aetiologies, especially erysipelas.

Few studies have been published on the prevalence of dermatological conditions seen in emergencies (Less than twenty worldwide). Our context is characterized by their high frequency. In fact, 2912 patients were seen during the period of two years, including 843 with a true dermatological emergency having a vital or a severe functional prognosis. The average was 29 patients per week, including 10 patients with poor prognosis. This prevalence related to time is on the same scale as that found by Dr Ziat at the University Hospital of Fez (204 cases in 6 months) [4]. However, it greatly exceeds the prevalence seen in the series of Ly *et al*. (162 cases in 10 months) [5], and Falanga *et al*. (971 cases over 5 years) [6], and remains lower than that seen in the series of Murr *et al*. (100 cases in 1 month) [7]. This finding can be explained by the health system organization of each country. Our system is less flexible in terms of access to consultations, and less vigilant in terms of access to emergencies. The percentage of true dermatological emergencies among all patients seen in the emergency unit is 28.95%, which is consistent with the findings of other series that varies from 18% to 43% [8-10].

The mean age of the patients was 46.95 years, in agreement with the findings of other studies ranging from 41 to 53 years [11-14]. However, unlike other studies where there was a majority of women [2, 14, 15], our series is characterized by a slight predominance of men (53.2%), as found by Bancalari-Díaz *et al*. (54.1%) [13]. The main source of referral was the central emergency unit (53.2%), as reported by Grillo *et al*. (57%) [16] and Gil Mateo *et al*. (86%) [17].

In foreign series, the most common reasons for consultation are drug-induced skin reactions [1, 2, 5, 6, 10] or inflammatory dermatoses [7]. However, in our Moroccan context and in an Iranian series [18], infectious dermatoses predominate. Indeed, the particularity of our series is the high frequency of infectious dermatoses reaching 55.63%, while it doesn´t exceed an average of 30% in developed countries' series [11, 19]. This can be explained by poorly balanced diabetic patients, non-respect of hygiene measures and difficulty to access health care structures. The majority of these patients didn´t require hospitalization; hence the important role of the general practitioner and the intern, who can also treat these dermatoses when there are no severity signs.

The hospitalization rate in our series is 27.88% (n=235), which is lower than the one in a Turkish series (34.5%) [1], but much higher than in the other series (2% to 5%) [2]. This huge difference is related to the absence of a daycare hospitalization unit in our department, which can handle a large part of activities, and to the fact that our study only included true emergencies.

Our study answered a key question: what are the dermatoses that trigger the emergency? It covered all dermatological emergencies, regardless of their class. However, its limitation was its retrospective nature and therefore the unavailability of some data.

## Conclusion

The pathologies seen in the dermatological emergency unit are very diverse. They are likely to lead to significant morbidity and mortality in the absence of optimal care in the first hours. However, the majority of the patients didn´t require emergency consultation. Therefore, there is a need to improve the non-dermatologist physicians' knowledge of emergency skin disorders. Taking into consideration the results of our study, it may be suggested to give more emphasis to infectious dermatoses in the medical program of our country, which would be beneficial both for the practitioner and patient care. Moreover, it is important to inform the population which type of dermatological presentations constitutes true emergencies to reduce the overcrowding of emergencies departments.

### What is known about this topic

- A true dermatological emergency can involve the patient's vital or functional prognosis;
- Dermatological emergencies are a real public health problem;
- Not all emergency department patients have a true dermatological emergency.

### What this study adds

- Infectious dermatoses are the most frequent reason for emergency dermatological consultations in Morocco;
- More than half of the patients accessing the dermatological emergency unit are referred from the central emergency department;
- Less than the third of the patients with a dermatological emergency required hospitalization.
